# The psychological impact of the COVID-19 outbreak among the fever patients in the lockdown zone

**DOI:** 10.1186/s40359-023-01113-6

**Published:** 2023-03-14

**Authors:** Yuewei Chen, Qian Ma, Xiaoshuai Xie, Kekui Cao, Zhencai Hou, Peiyi Zhang

**Affiliations:** 1 The Department of Disease Control and Prevention, The Armed Police Forces Hospital of Shandong, Jinan, 250000 China; 2 First Department of Cardiology, First Affiliated Hospital, School of Medicine, Shihezi University, Shihezi, 832008 Xinjiang China; 3 Department of Urology, Shandong Provincial Hospital Affiliated to Shandong First Medical University, Jinan, 250000 China; 4 Department of Rheumatology and Immunology, Jinan Central Hospital Affiliated to Shandong University, Jinan, 250000 China

**Keywords:** Psychological impact, COVID-19, Fever patients

## Abstract

**Background:**

COVID-19 pandemic is still ongoing, which not only impact physical health but psychological health. This research aims to analyze the psychological impact of residents with a fever (> 37 °C) during the COVID-19 outbreak in one community.

**Methods:**

There were 105 participants surveyed online from 7th March to 21st March 2022. Collected the data included the socio-demographics, health status, COVID-19 knowledge and concerns and the Impact of Events Scale-Revised (IES-R) ratings.

**Results:**

Among those participants, the IES-R mean score was 24.11 (SD = 6.12), and 30.48% of respondents reported a moderate to the severe psychological impact. Female gender; youth age; single status; other specific symptoms; concerns about family members, and discrimination were significantly associated with the greater psychological impact of the COVID-19 event (*p* < 0.05).

**Conclusions:**

In the lockdown zone, about one-third of the residents have an obvious psychological impact after fever. The factors identified can be used to make effective psychological support strategies in the early stages of the COVID-19 outbreak.

## Introduction

The COVID-19 pandemic is still ongoing which caused a serious impact on individuals globally at any age, and ethnicity [1]. This infectious disease has led to a high mortality rate and morbidity around the world [2, 3]. It not only affects physical health but has a negative impact on psychological health like increasing the rates of anxiety, and depression [4, 5]. The fear of sickness or infection with the new coronavirus, helplessness, and anxiety due to isolation also lead to the spread of public mental health and psychological crises which meaning symptoms related to discomfort and distress or worse, such as anxiety or panic attacks [6].

Fever is one of the most common symptoms in COVID-19 patients which have been aroused the great attention of the public. Now in China, the government has established lots of specialized fever clinics to screen COVID-19 patients. So patients with fever will be given lots of attention and they will experience more surveys like the history of the epidemic, nucleic acid screening or isolated observation. Those in quarantine might experience boredom, loneliness, and anger [7].

The community is the main activity area for residents, which would be divided into lockdown zones if one COVID-19 case was found, then it would cause outbreak events. So the requirements for health management are more strict in the community. Once there is a patient with a fever, he/she as a key healthcare object will be isolated alone. During the quarantine, such patients will bear psychological pressure, which will affect the treatment effect but also cause certain psychological pressure on the patient’s family. For this reason, we conducted the present research with the aim of analyzing the psychological impact among fever patients in the lockdown zone and identifying risk factors contributing to the psychological crisis.

## Methods

### Study design, setting and participants

The present study was performed from 7th March 2022 to 21st March 2022, All the patients in one community which was divided into lockdown zone in China during the outbreak of COVID-19, and cross-sectional analysis in The Armed Police Forces Hospital of Shandong and First Affiliated Hospital, School of Medicine, Shihezi University. There were 105 residents who had a high temperature (> 37 °C) were selected as the objects. Before participation, experimental procedures were explained to all the participants, who gave their voluntary written informed consent. The entire research procedure was conducted online. None of the participants had previous or recent experience in these processes.

### Assessments

The survey included information on socio-demographics, personal symptoms, knowledge and concerns about COVID-19, and the Impact of Events Scale-Revised (IES-R) instrument [8, 9]. The Impact of Event Scale-Revised (IES-R) has 22 items. This 22-item scale is factorized of three dimensions, namely: intrusion with eight items; avoidance with eight items; and hyperarousal with six items. The IES-R is designed with five Item Response Anchors rated from 0 to 4, where 0 indicates not at all; 1 = a little bit; 2 = moderately; 3 = quite a bit; and 4 = extremely. Subsequently, scores on the 22-item IES-R range from 0 to 88. Higher scores are interpreted as having more severe impact.

### Statistical analysis

The statistical analysis was conducted using SPSS version 21.0 for Windows (SPSS Inc., Chicago, IL, USA). Frequency and percentage were applied to describe variables. The scores of IES-R were expressed as mean and standard deviation (SD). Association analysis using chi-square test. *p* value < 0.05 was considered statistically significant. We used the logistic regression models to analyse which were the influence factors for the psychological reaction. For the logistic regression models, the total score of the scale was treated as a dichotomous categoric variable accounting for either normal and mild psychological impact (score below the cutoff, i.e., IES-R < 33) or severe psychological impact (score equal or higher of the cut-off, i.e., IES-R > 33) [9].

## Results

The baseline characteristics of participants were presented in Table 1. And 105 participants were enrolled in the present study, among the enrolled patients, 62 (59.04%) patients were male and 43 (40.96%) patients were female, well-educated (12.38%, at least a bachelor’s degree), single status (36.19%) and members of the household size of 3–4 people (44.76%). The mean age of respondents was 24 years (SD, 2. years).

**Table 1:** Demographic variables and association with psychological impact among the fever patients (n = 105)

Variable	n (%)	Psychological impact		*p* value*
		Normal and mild n (%)	At least moderate n (%)	
Gender				< 0.001
Male	62 (59.04)	42 (67.74)	20 (32.26%)	
Female	43 (40.96)	20 (46.51)	12 (53.49)	
Age (years)				< 0.001
12–20	34 (32.38)	19 (55.88)	15 (44.12)	
21–24	45 (42.86)	37 (82.22)	8 (17.78)	
> 24	26 (24.76)	17 (65.38)	9 (34.62)	
Educational attainment				0.026
High school and lower	24 (22.86)	15 (62.50)	9 (37.50)	
College	68 (64.76)	49 (72.06)	19 (27.94)	
Bachelor higher	13 (12.38)	9 (69.23)	4 (30.77)	
Census register				0.164
Village	43 (40.95)	30 (69.77)	13 (30.23)	
City	62 (59.05)	45 (72.58)	19 (27.42)	
Marital status				< 0.001
Single	38 (36.19)	20 (52.63)	15 (42.37)	
Married	67 (63.81)	50 (74.63)	17 (25.37)	
Health care professional				0.023
Yes	43 (27.62)	32 (74.42)	11 (25.58)	
No	62 (72.38)	41 (66.13)	21 (33.87)	
Household size				0.061
2 persons or few	33 (31.43)	22 (66.67)	11 (33.33)	
3–4 persons	47 (44.76)	30 (63.83)	17 (36.17)	
5 persons or more	25 (23.81)	18 (63.83)	17 (36.17)	
Whether is a local resident				< 0.001
Yes	78 (74.28)	57 (73.08)	21 (26.92)	

The psychological impact was measured using the IES-R scale, which revealed a sample mean score of 24.11 (SD, 6.12). Of all respondents, there were 23 (21.90%) reported minimal psychological impact (score: 0–23); 50 (47.62%) rated mild psychological impact (score: 24–32); and 32 (30.48%) reported a moderate to severe psychological impact (score: > 33). The male respondents had significantly lower scores in IES-R (*p* < 0.05) compared to females. The patients in the young age group (< 20 years) and the single group had significantly high IES-R scores (*p* < 0.05). The non-healthcare professionals had significantly higher IES-R scores (*p* = 0.023) than healthcare professionals. Respondents who had a higher level of education (Bachelor’s) had significantly lower IES-R scores (*p* = 0.026). Respondents who were not local residents had higher IES-R scores (*p* < 0.05).

The Physical health status and association with psychological impact are in Table 2. There were 20.96% of the respondents had a fever at least 3 days within the 1 week before the survey with higher IES-R scores (*p* = 0.022) and 37.14% had a high fever with a body temperature over 38 °C, and there were other respondents reported headache (71.43%), cough (56.19%), breathing difficulty (45.71%), sore throat (65.71%), recent testing for COVID-19 in the past 7 days (90.48%) These symptoms were significantly associated with higher scores for IES (*p* < 0.05).

**Table 2:** Physical health status and association with psychological impact among the fever patients (n = 105)

Variable	*n* (%)	Impact of event		*p* value*
		Normal and mild *n* (%)	At least moderate *n* (%)	
Initial body temperature				0.043
37–38 °C	66 (62.86)	43 (80.30)	13 (19.70)	
> 38 °C	39 (37.14)	20 (51.28)	19 (48.72)	
Duration of fever (days)				0.022
< 1	18 (17.14)	14 (77.78)	4 (22.22)	
2	65 (61.90)	47 (72.31)	18 (27.69)	
≥ 3	22 (20.96)	12 (54.45)	10 (45.55)	
Chills				0.068
No	66 (62.86)	46 (69.70)	20 (30.30)	
Yes	39 (37.14)	27 (69.23)	12 (30.77)	
Headache				0.034
No	30 (28.57)	20 (66.67)	10 (33.33)	
Yes	75 (71.43)	53 (70.67)	22 (29.33)	
Body pain				< 0.001
No	33 (31.43)	21 (63.64)	12 (36.36)	
Yes	72 (68.57)	52 (72.22)	20 (27.78)	
Cough				< 0.001
No	46 (43.81)	35 (76.09)	11 (23.91)	
Yes	59 (56.19)	38 (64.41)	21 (35.59)	
Breathing difficulty				0.024
No	57 (54.29)	44 (77.19)	13 (22.80)	
Yes	48 (45.71)	29 (60.42)	19 (39.58)	
Dizziness				0.057
No	43 (40.95)	29 (67.44)	14 (32.56)	
Yes	62 (59.05)	44 (70.97)	18 (29.03)	
Sore throat				< 0.001
No	36 (34.29)	29 (80.56)	7 (19.44)	
Yes	69 (65.71)	44 (63.77)	25 (36.23)	
Consultation with a doctor in the clinic in the past 14 days				0.065
No	26 (24.76)	17 (65.38)	9 (34.62)	
Yes	79 (75.24)	56 (70.89)	23 (29.11)	
Recent testing for COVID-19 in the past 7 days				< 0.001
No	10 (9.52)	4 (40)	6 (60)	
Yes	95 (90.48)	69 (72.63)	26 (27.37)	

Concerns about COVID-19 and its association with psychological impact in Table 3. Most respondents (90.48%) knew that the routes of transmission of the virus COVID-19 were airborne, respectively. Information was mainly sourced from social media and the internet by 71.43% of the respondents. The proportion of respondents who had confidence in their own doctors’ ability to recognize COVID-19 was 83.81%. About 46.61% of participants felt they will likely infect with COVID-19 during the outbreak if they had a fever. The proportion of respondents who felt discriminated by other people was 24.76%.

**Table 3:** Concern and association with psychological impact among the fever patients (n = 105)

Variable	*n* (%)	Impact of event		*p* value*
		Normal and mild *n* (%)	At least moderate *n* (%)	
Contact via contaminated objects				0.135
Agree	90 (85.71)	64 (70)	27 (30)	
Disagree	10 (9.52)	7 (70)	3 (30)	
Don’t know	5 (4.77)	3 (60)	2 (40)	
Airborne				0.207
Agree	95 (90.48)	66 (60.47)	29 (30.53)	
Disagree	3 (2.86)	2 (66.67)	1 (33.33)	
Don’t know	7 (6.66)	5 (71.43)	2 (28.57)	
Satisfaction with the amount of health information available about COVID-19				< 0.001
Very satisfied	73 (69.52)	55 (75.34)	18 (24.66)	
Satisfied	15 (14.29)	9 (60)	6 (40)	
Dissatisfied	10 (9.52)	6 (60)	4 (40)	
Very dissatisfied	7 (6.67)	3 (42.86)	4 (57.14)	
Number of cases infected by COVID-19				0.068
Heard	90 (85.71)	63 (70)	27 (30)	
Not Heard	15 (14.29)	10 (66.67)	5 (33.33)	
Number of recovered cases infected by COVID-19				0.073
Heard	95 (90.48)	66 (69.47)	29 (30.53)	
Not Heard	10 (9.52)	7 (70)	3 (30)	
The main source of health information				0.095
Social media and internet	75 (71.43)	55 (73.33)	20 (26.67)	
Traditional media	15 (14.29)	9 (60)	6 (40)	
Family members	12 (11.43)	7 (58.33)	5 (41.67)	
Others	3 (2.85)	2 (66.67)	1 (33.33)	
Level of confidence in own doctor’s ability to diagnose or recognize COVID-19				0.003
Very confident	58 (55.24)	48 (82.76)	10 (17.24)	
Somewhat confident	22 (20.95)	11 (59.09)	9 (40.91)	
Not very confident	12 (11.43)	6 (50)	6 (50)	
Not at all confident	5 (4.76)	2 (40)	3 (60)	
Do not know	8 (7.62)	4 (50)	4 (50)	
Concerns about other family members getting COVID-19				< 0.001
Very worried	48 (45.71)	34 (70.83)	14 (29.17)	
Somewhat worried	33 (31.43)	21 (63.64)	12 (36.36)	
Not very worried	15 (14.29)	11 (73.33)	4 (26.67)	
Not worried at all	9 (8.57)	7 (77.78)	2 (22.22)	
The feeling of being discriminated against by other people				< 0.001
Yes	26 (24.76)	17 (65.38)	9 (34.62)	
No	79 (75.24)	56 (70.89)	23 (29.11)	

We found there was an interesting phenomenon that the high satisfaction with the amount of health information available about COVID-19 was associated with low IES-R scores (*p* < 0.001). Very confidence in a doctor’s ability to diagnose or recognize COVID-19 was associated with low IES-R scores (*p* = 0.003).

Logistic regression analyses for psychological impact at least of moderate level in Table 4. We performed logistic regression analyses to analyse the factors that cause patients to be more prone to the psychological stress response. The patients with age lowed 20 years, female sex and who was not healthcare professional was more suffered a moderate level of psychological impact (*p* < 0.05).

**Table 4:** Logistic regression analyses for psychological impact at least of moderate level

Factor		β	SE	Wald χ^2^	*p* value	OR (95% CI) adjusted
Age	< 20	0.57	0.32	6.11	0.01	1.38 (1.13–3.39)
Sex	Female	0.27	0.22	5.24	0.03	1.27 (1.15–2.35)
Health care professional	No	0.34	0.09	13.14	< 0.01	1.41 (1.17–1.70)

## Discussion

Fever is the typical symptom of COVID-19 patients, and it had aroused great concern for the government in China. The community will be divided into the lockdown zone if there appeared COVID-19 patients, which will get stricter management [10]. The residents in the lockdown zone would be more inclined to anxiety than other communities and face more stress if they had at least one symptom compared to the COVID-19 patients [11]. This survey was conducted in the first month when COVID-19 was broken out, and enhanced community quarantine was implemented in one community in China in 2022. In this research, we surveyed a total of 105 residents who had a fever (> 37 °C) but had not been diagnosed with COVID-19. There were 30.48% of the respondents reported moderate to a severe psychophysiological stress response. Since the high mortality rate and disability rate during COVID-19, the prevalence of severe psychophysiological stress response is still increasing with the increasing trend of confirmed cases in many studies [12, 13]. In the present study, we noticed that this level was higher than in the other studies, indicating the effect on psychology among patients with fever was more obvious than among general people. We found females were more affected than males. Younger, less educated, single people, not local residents reported a more obvious psychological impact. These subgroups, considered at higher risk for developing adverse psychological outcomes, may get low social and emotional support once they were isolated, which will increase the chance of feelings of fear, and isolation [14].

The respondents with higher temperatures or longer fevers lasting were more tend to have stress on psychology. The longer the symptoms last, the longer quarantine periods will be demanded, which means the respondents would arouse more attention and cause greater psychological stress [15]. The respondents with cough, headache, breathing difficulty, and sore throat, which were more affected by psychological health according to reports from China and the Philippines [16, 17]. Those common phenomena were more obvious in COVID-19 patients, so the respondents would be more fearful and anxious about the COVID-19 event [18]. Many patients with those symptoms would suspect that they had been infected with a novel coronavirus that will make a heavy burden on their psychology. Besides that, with more symptoms, they would get more attention no matter from their family or the medical worker. That will make a deep imply that they had more possibility to be a COVID-19 patient.

The fever respondents with a positive view of COVID-19 would be more possible to assess their health correctly and be more confident that they would not be the COVID-19 patient. More knowledge about COVID-19 will help the respondents take effective measures to protect themself and lowdown the risk of being a COVID-19 patient [19, 20]. Others lacked the knowledge and cannot rightly analyse their health status once they heard there was someone who got COVID-19 which will heighten their suspicion of others and make them more anxious about the external environment [21, 22]. Most respondents were confident in their doctors’ abilities. The confidence seemed to be protective against negative mental health states and lower levels of stress. The respondents maybe also were affected by the others' health. In this research, there were 78.14% of respondents felt worried when other family members got COVID-19. During the pandemic, the individual would pay more attention to the report about the COVID-19 event, especially the residents in quarantine [23]. Once there was one report about there increased new COVID-19 cases in the quarantine, the respondents would improve their vigilance giving them great mental stress like changing their sleeping and normal eating habits [24]. Some negative information about COVID-19 would also cause deeper harmful effects for the residents of quarantine, like the Increasing incidence of depression and suicide [25, 26]. The respondents who felt discriminated against by others will be more likely to cause psychological stress than others, which would make a harmful effect on their health and increase the incidence of other diseases like depression and bipolar disorder [27].

In the lockdown zones, the residents were restricted to a small area which is stressful as it prevents face-to-face connections and traditional social interactions [28]. For the fever patient who would be subjected to further quarantine, might be more fears of the infection spreading among family members, frustration and boredom from being isolated [29].

The present study showed that females and younger age reported psychological impact during the pandemic. This is in line with a review and meta-analysis by Serrano-Ripoll et al. [30], which identified female sex, younger age, lack of support, stigma and occupational parameters as risk factors for mental health deterioration during epidemic outbreaks. One of the few China studies conducted in Hubei also found that females reported more severe symptoms of depression, anxiety, and distress [31]. A possible explanation for this may be provided by the fact among the nurses most of them were female, they had direct and longer contact with patients, except that the female would face more duties including work or family.

Another finding of the present was that healthcare professionals exhibited low IES-R scores which were different from the studies in Italy and Spain. Giusti E.M. found that front-line healthcare workers had a higher risk of symptoms of depression [32]. This difference may be attributed to the fact that in this Italian study, the majority of participants were employed in COVID-19 units and thus directly confronted with the COVID-19 disease. In this study, the healthcare professionals were quarantined at home after they had a fever, and they could assess their health more properly than the other patients.

The present study has several strengths. First, it is the first time the association between psychological impact and normal fever people was discovered during the COVID-19 epidemic, Second, the demographic in normal fever patients was documented, which conveyed valuable information on following studies to screen new risk factors for psychological impact in special era. Thirdly, this study is a cross-sectional study with community as the unit and has good representativeness. Our study has several limitations. First, the survey was done online and lacked effective guidance. Some respondents may not answer the questionnaire truly and accurately. Second, the survey was implemented in the early stage of the COVID-19 event and the respondents may change their psychological outcomes throughout the public health crisis.

## Conclusions

During the early phase of the COVID-19 outbreak in the lockdown zone, one-third of the respondents reported a moderate-to-severe psychological impact of the outbreak. Female gender, youth age of 12–20 years, single status, lower level of education, not a local resident, presence of specific physical symptoms (i.e., headache, cough), dissatisfied with the health information about COVID-19, worry about family members getting COVID-19, being discriminated by others This study will be used to make appropriate measures psychological to avert the occurrence of mental health problems preventing psychological crisis on the early stage during the outbreak of COVD-19.

## Data Availability

The data sets generated and/or analysed during the current study are not publicly available due to the necessity to ensure participant confidentiality policies and laws of the country but are available from the corresponding author upon reasonable request.
